# Assessment of the Novel, Practical, and Prognosis-Relevant TNM Staging System for Stage I-III Cutaneous Melanoma

**DOI:** 10.3389/fonc.2022.738298

**Published:** 2022-04-29

**Authors:** Di Hu, Zeming Liu, Sichao Chen, Yihui Huang, Wen Zeng, Wei Wei, Chao Zhang, Ling Zhou, Danyang Chen, Yiping Wu, Liang Guo

**Affiliations:** ^1^Department of Plastic Surgery, Zhongnan Hospital of Wuhan University, Wuhan, China; ^2^Department of Plastic Surgery and Cosmetic, Tongji Hospital, Tongji Medical College, Huazhong University of Science and Technology, Wuhan, China; ^3^Department of Ophthalmology, Zhongnan Hospital of Wuhan University, Wuhan, China; ^4^Department of Pediatrics, St John Hospital and Medical Center, Detroit, MI, United States; ^5^Department of Cardiovascular Surgery, Union Hospital, Tongji Medical College, Huazhong University of Science and Technology, Wuhan, China

**Keywords:** cutaneous melanoma, staging system, AJCC, SEER, prognosis

## Abstract

**Background:**

The clinical TNM staging system does not differ between the 7^th^ and 8^th^ editions of the American Joint Committee on Cancer (AJCC) staging manual. A more practical TNM staging system for patients with stage I-III cutaneous melanoma are needed.

**Methods:**

Data were accessed from the Surveillance, Epidemiology, and End Results (SEER) open database. We divided the patients into 32 groups based on the T and N categories. The Kaplan-Meier survival curves and treatment guidelines were used to proposed a new TNM staging system. Cox proportional hazards model and 1000-person-years were used to verify accuracy.

**Results:**

This retrospective study included 68 861 patients from 2010 to 2015. The new proposed staging system was as follows: stage IA, T1aN0M0; stage IB, T1b/T2aN0M0; stage IIA, T3-4aN0M0 and T2bN0M0; stage IIB, T1-4aN1-2M0 and T3-4bN0M0; and stage III, T1-4aN3M0 and T1-4bN1-3M0. Hazard ratios for the new stages IB, IIA, IIB, and III, with stage IA as reference, were 4.311 (95% confidence interval [CI]: 3.217-5.778), 8.993 (95% CI: 6.637-12.186), 13.179 (95% CI: 9.435-18.407), and 20.693 (95% CI: 13.655-31.356), respectively (all p-values < 0.001). Cancer-specific mortality rates per 1000-person-years were 0.812 (95% CI: 0.674-0.978), 6.612 (95% CI: 5.936-7.364), 22.228 (95% CI: 20.128-24.547), 50.863 (95% CI: 47.472-54.496) and 120.318 (95% CI: 112.596-128.570) for stages IA, IB, IIA, IIB and III, respectively.

**Conclusion:**

We developed a more practical and prognosis-relevant staging system than that of the 8^th^ edition AJCC manual for patients with stage I-III cutaneous melanoma. Treatments using this new model would improve the quality of life and survival rates of patients with melanoma.

## Introduction

Melanoma, a malignant tumor arising from melanocytes, is linked to ultraviolet exposure and severe episodic sunburn early in life correlates with melanoma risk (1, 2). The incidence of melanoma has increased over the last several decades, rising at a rate of 3–7% on average and rising particularly faster among Caucasian men and the elderly (3). In the United States, approximately 91, 270 new cases of cutaneous melanoma, which is considered to be the fifth most common cancer in men and the sixth most common in women, were reported in 2018 (4). Another increase in the incidence of melanoma is projected without signs of leveling-off in Australia, Germany, and other countries (5–7). Thus, the diagnosis, treatment, and prognosis of melanoma have raised comprehensive attention.

Compared with the 7^th^ edition of the American Joint Committee on Cancer (AJCC) manual, the clinical 8^th^ edition introduced some key changes: 1) definitions of T1a and T1b have been revised as <0.8 mm without ulceration and 0.8‐1.0 mm with or without ulceration, and <0.8 mm with ulceration; 2) mitotic rate is no longer considered as a T category criterion; and 3) descriptors have been added to each M1 subcategory designation for lactate dehydrogenase (LDH) level (LDH elevation no longer upstages M1c) (8). However, there is no change in clinical TNM staging between the 7^th^ and 8^th^ edition (8, 9).

The objectives for updating the AJCC staging manual are guiding patient treatment, providing better estimates of prognosis, and refining the stratification of patients who enter clinical trials (10). Thus, this study investigated the appropriate staging process in patients based on the 8^th^ edition and cancer-specific mortality of patients with melanoma, thus providing a practical clinical staging system for the early diagnosis of melanoma in these patients.

## Methods

### Patients and Database

For this study, we collected data of patients with melanoma (code: 8720/3-8723/3, 8730/3, 8740/3-8746/3, 8761/3, 8770/3-8774/3, 8780/3) between 2010 and 2015 from the openly accessible Surveillance, Epidemiology, and End Results (SEER) Program of the National Cancer Institute, which is an authorized source of information on cancer incidence and survival in the United States. There was no ethical review required because SEER is a publicly available database with anonymized data.

In this study, 11,641 cases with recorded categories of T0, TX, and NX were excluded. Furthermore, a status described as “T1NOS” and “T2NOS” does not exist in the TNM criteria defined in the AJCC TNM staging system; therefore, we excluded such cases. Patients with distant metastasis were also excluded in this study for the following two reasons. First, patients with M1 were classified as stage IV owing to its unfavorable prognosis, and second, only 183 cases were seen, which is a small number for statistical analysis.

After filtering the data, 68, 861 cases were included in this study. Information in relation to age at diagnosis, sex, race, year of diagnosis, T and N categories, histology, thickness, ulceration, mitotic index, sentinel lymph node metastasis, extension, radiation, chemotherapy, and surgical method were also collected. Missing or unclear data were treated as user missing values.

### Development Process

The patients were divided into 32 groups according to the T and N categories. These groups were then divided into five stages based on the trends of the Kaplan-Meier (K-M) survival curves. The group classifications were further adjusted according to the treatment guidelines and the 8^th^ edition of the AJCC staging manual. Furthermore, the probability of mortality per 1000-person-years and Cox proportional hazards models were used to assess the prognosis of the patients at different stages. The results of the Cox analysis were adjusted for age at diagnosis, sex, race, year of diagnosis, histology, thickness, ulceration, mitotic index, selected lymph node meets, extension, radiation, chemotherapy, and surgical method.

### Statistical Analysis

Variables are summarized as frequencies, proportions, and mean values ± standard deviations, as appropriate. We used the following statistical methods of survival analyses in the modelling process: K-M curves, Cox proportional hazards models, and mortality per 1000-person-years. To evaluate the predictive ability and accuracy of this model, we set age at diagnosis, sex, race, year of diagnosis, histology, thickness, ulceration, select lymph node, mitotic rate, tumor size, extension, proposed stage, radiation, chemotherapy, and surgery as covariates, and melanoma specific mortality as dependent to calculate predicted probability (PRE-1) and Hosmer-Lemeshow goodness-of-fit, which used to calculate the sum of receiver operating characteristics (ROC) curve and calibration curve. P ≤ 0.05 was considered statistically significant. All statistical analyses were performed using SPSS, version 22.0 (IBM Corp., Armonk, NY, USA), Stata/SE version 15 (Stata Corp, College Station, TX, USA), GraphPad Prism version 7 (GraphPad Software Inc., La Jolla, CA, USA), or MATLAB version 2018a (MathWorks, Cambridge University Press, Cambridge, UK).

## Results

Patient DemographicsDemographic data, clinical characteristics, and treatment methods of the patients are summarized in **Supplementary Table 1**. The mean age of the 68,861 patients was 58.48 (± 16.20) years. Furthermore, 49, 773 patients were aged ≥ 50 years. The approximate sex ratio was 1. Among the 68, 861 patients, 41, 141 (59.75%) has T1a diseases. For N categories, 64, 059, 2, 732, 1, 399, and 671 patients has N0, N1, N2, and N3 disease, respectively.

### The Proposed TNM Staging System

**Figure 1:**
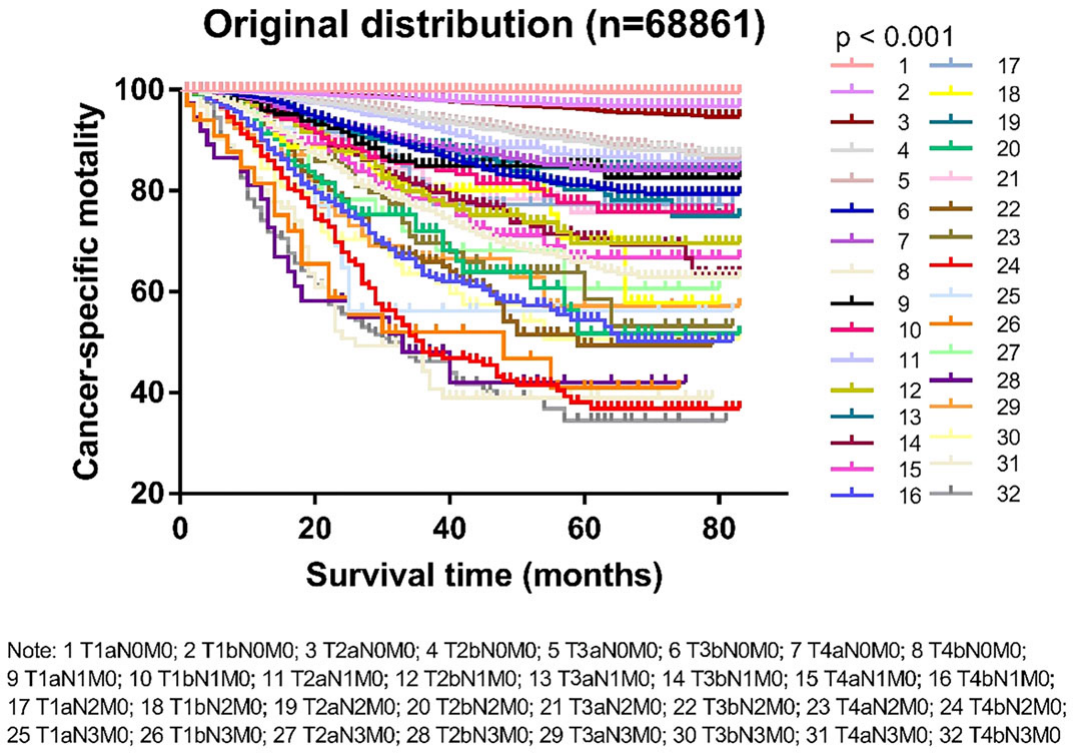
Kaplan-Meier survival curves in patients with cutaneous melanoma divided into 32 groups by T and M categories.

Based on the T and N categories, the patients were divided into 32 groups (**Supplementary Table 2**). The survival status of the different groups is shown in **Figure 1**. According to the survival trends, we divided the 32 groups into five stages, named *Adjusted Distribution*, as follows: stage I, T1-2aN0M0 and T1bN0M0; stage IIA, T3-4aN0M0 and T2bN0M0; stage IIB, T3-4aN1M0, T1-4aN2M0, and T3-4bN0M0; stage IIC, T1-4aN3M0, T1-4bN1M0, and T1-4bN2M0; and stage III, T1-4bN3M0 (**Table 1**).

**Table 1:** Comparison of different distribution of 32 groups.

Code	Groups	Adjusted distribution	New proposed
1	T1aN0M0	T1-2aN0M0	T1aN0M0
2	T1bN0M0	T1bN0M0	
3	T2aN0M0		T1bN0M0
4	T2bN0M0		T2aN0M0
5	T3aN0M0		
6	T3bN0M0	T3-4aN0M0	T3-4aN0M0
7	T4aN0M0	T1-2aN1M0	T2bN0M0
8	T4bN0M0	T2bN0M0	
9	T1aN1M0		
10	T1bN1M0	T3-4aN1M0	T3-4bN0M0
11	T2aN1M0	T1-4aN2M0	T1-4aN1-2M0
12	T2bN1M0	T3-4bN0M0	
13	T3aN1M0		
14	T3bN1M0	T1-4aN3M0	T1-4aN3M0
15	T4aN1M0	T1-4bN1M0	T1-4bN1-3M0
16	T4bN1M0	T1-4bN2M0	
17	T1aN2M0		
18	T1bN2M0	T1-4bN3M0	
19	T2aN2M0		
20	T2bN2M0		
21	T3aN2M0		
22	T3bN2M0		
23	T4aN2M0		
24	T4bN2M0		
25	T1aN3M0		
26	T1bN3M0		
27	T2aN3M0		
28	T2bN3M0		
29	T3aN3M0		
30	T3bN3M0		
31	T4aN3M0		
32	T4bN3M0		

Adjusted distribution, classified by the survival trends; new proposed, based on adjusted distribution and consider of clinical experience.

**Figure 2:**
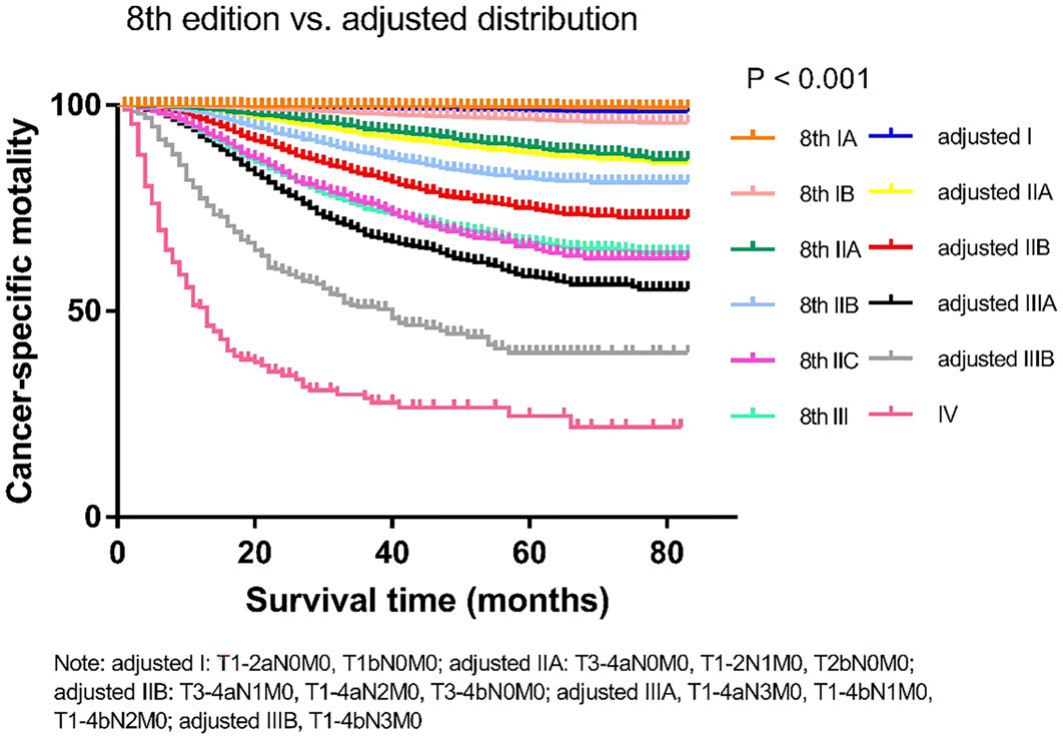
Comparison of the Kaplan-Meier curves of the 8^th^ edition of AJCC staging system and adjusted distribution edition based on data of cancer specific mortality.

**Figure 3:**
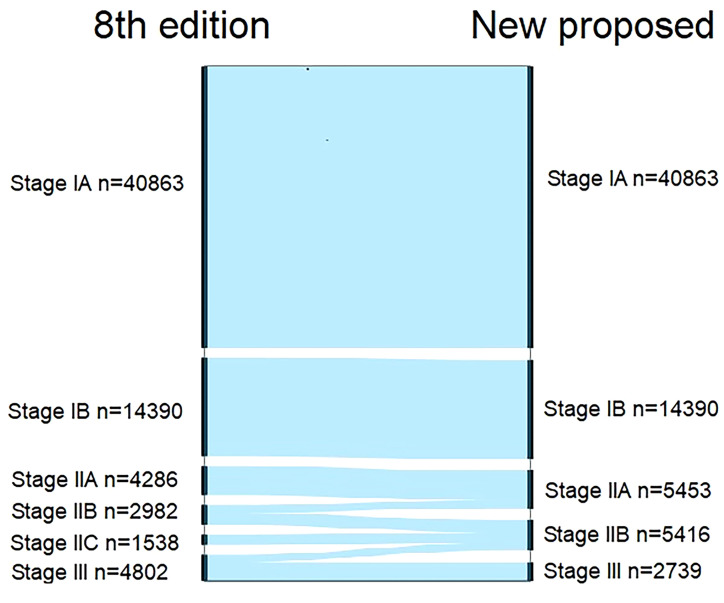
Alluvial flow diagram representing restaging of patients in the Surveillance, Epidemiology, and End Results database from the 8^th^ edition of AJCC staging system to the new proposed staging system.

We compared the cancer-specific survival trends of the condition classified based on the 8^th^ edition and the *Adjusted Distribution* in **Figure 2**. Considering the treatment guidelines, we proposed the development of a new staging system: stage IA, T1aN0M0; stage IB, T1b/T2aN0M0; stage IIA, T3-4aN0M0 and T2bN0M0; stage IIB, T1-4aN1-2M0 and T3-4bN0M0; and stage III, T1-3aN3M0 and T1-4bN1-3M0 (**Table 1**). In addition, a comparison of the 8^th^ edition and new proposed staging system is shown in **Figure 3** and **Table 2**.

**Table 2:** Comparison of the differences in distribution of patients with cutaneous melanoma between the 8^th^ edition and the new proposed TNM staging system.

Stages	8^th^ edition	No. (fail)	New proposed	No. (fail)
IA	T1aN0M0	40863 (111)	T1aN0M0	40863 (111)
IB	T1b/T2aN0M0	14390 (331)	T1b/T2aN0M0	14390 (331)
IIA	T2b/3aN0M0	4286 (273)	T3-4aN0M0, T2bN0M0	5453 (392)
IIB	T3b/T4aN0M0	2982 (332)	T1-4aN1-2M0, T3-4bN0M0	5416 (810)
IIC	T4bN0M0	1538 (317)	–	–
III	angTN1-3M0	4802 (1156)	T1-4aN3M0, T1-4bN1-3M0	2739 (876)

### Predictive Ability of the Newly Proposed TNM Staging System

**Figure 4:**
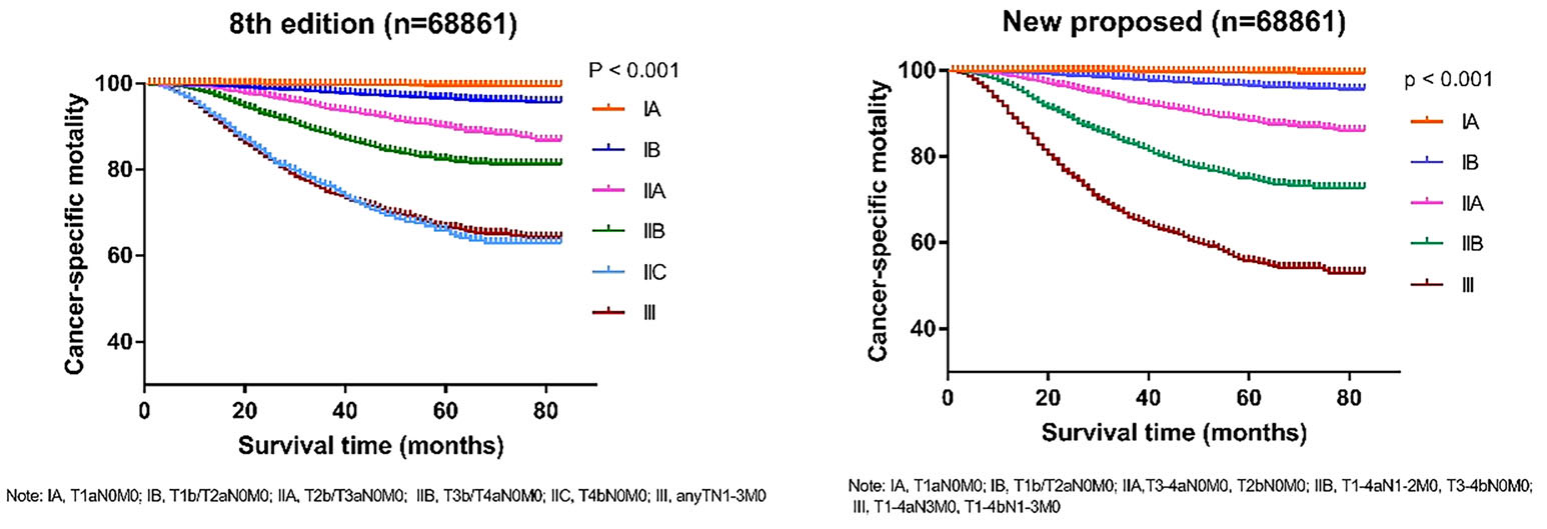
Comparison of Kaplan-Meier survival curves based on the 8^th^ edition of AJCC staging system and the new proposed staging system with the data of cancer-specific survival; T, tumor; N, node; M, metastasis.

**Figure 5:**
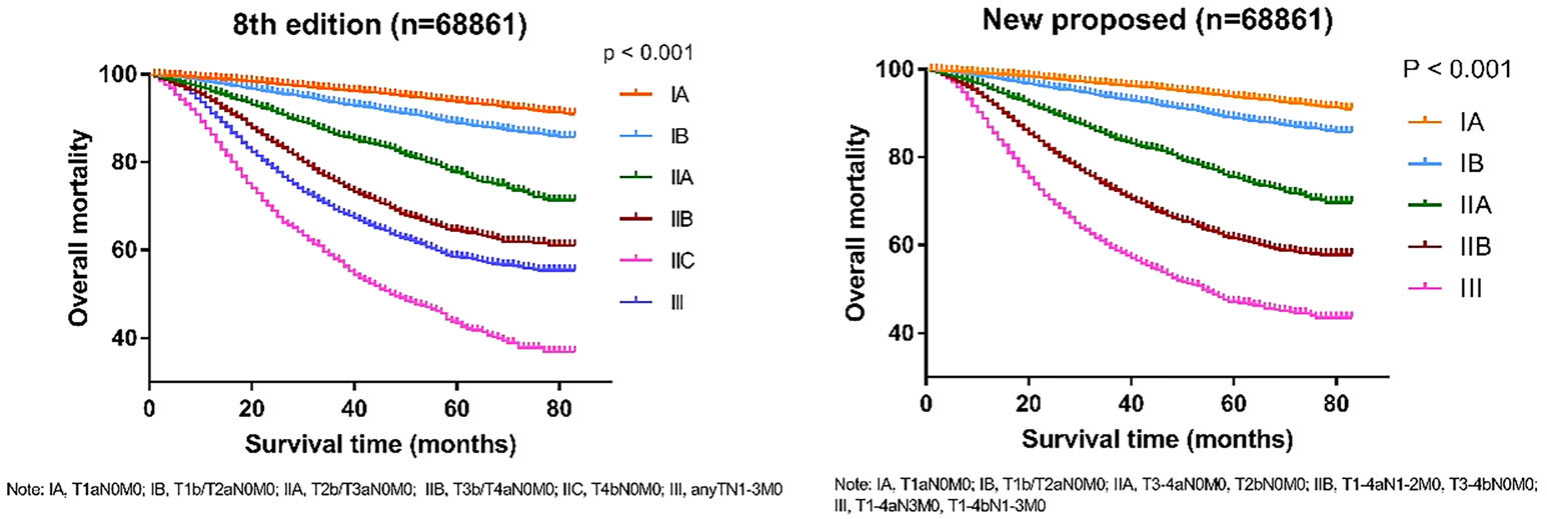
Comparison of Kaplan-Meier survival curves based on the 8^th^ edition of AJCC staging system and the new proposed staging system with the data of overall mortality; T, tumor; N, node; M, metastasis.

To verify the accuracy of the newly proposed TNM staging system, we formatted the K-M scores to estimate the rates of melanoma-specific survival (MSS), and overall survival (OS) generated from data stratified according to the 8^th^ edition of the AJCC and our newly proposed staging system (**Figures 4**, **5**), respectively. Compared with the 8^th^ edition, the survival trends were more distinguishable in the newly proposed TNM staging system.

**Figure 6:**
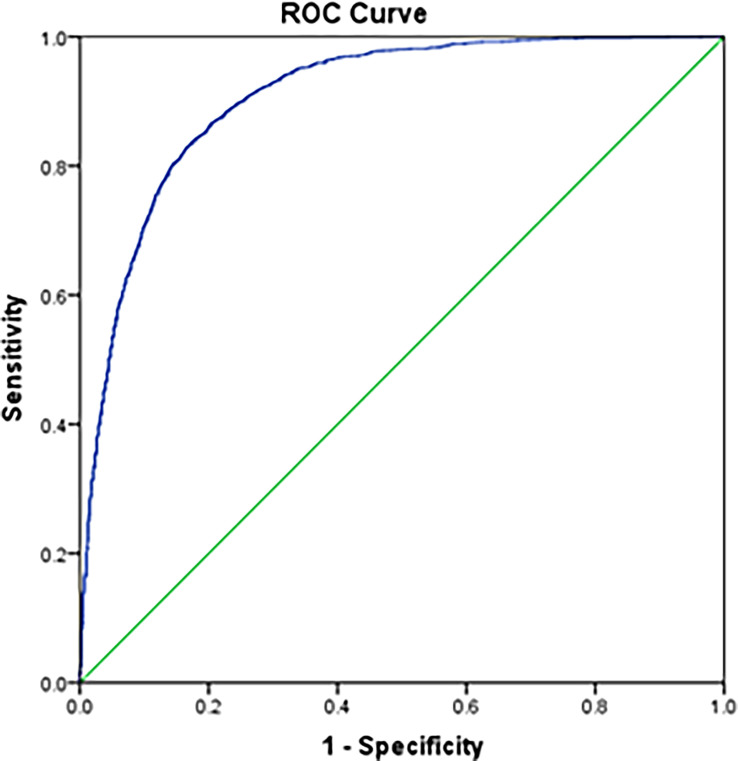
Receiver operating characteristics (ROC) curve of the new proposed staging system.

**Figure 7:**
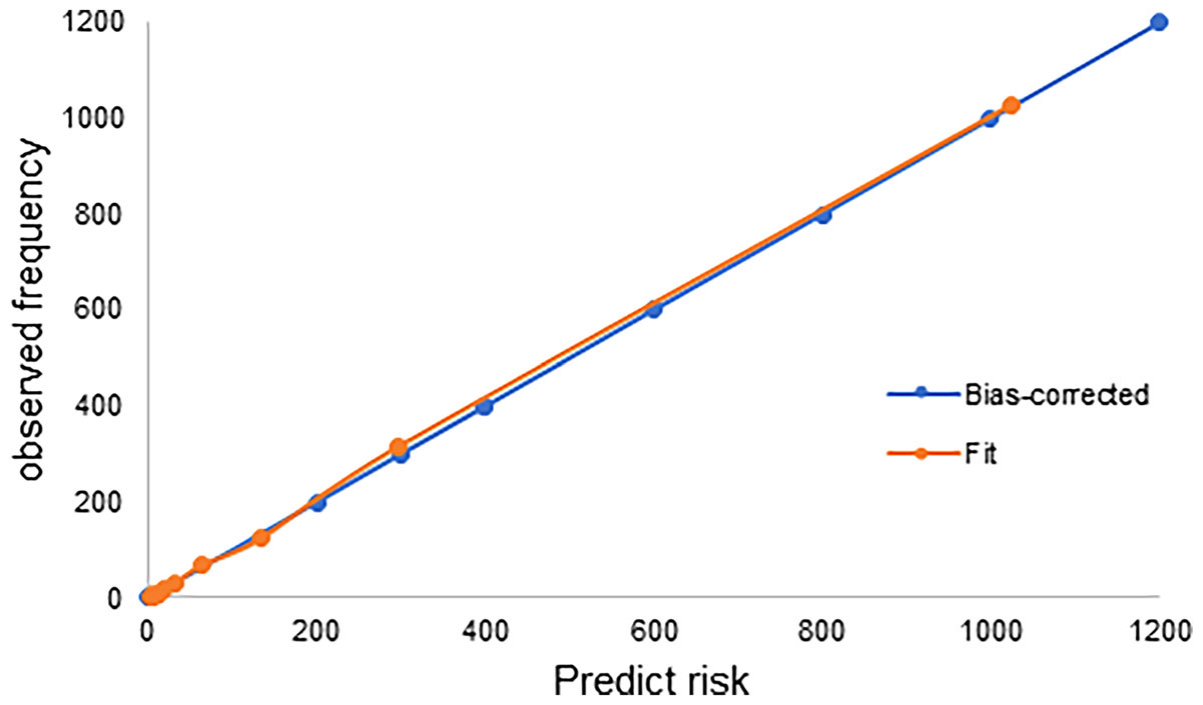
Calibration curve based on Hosmer and Lemeshow Test for the new proposed staging model.

**Table 3** shows the comparison between the survival status of patients in different stages using Cox analyses according to the 8^th^ edition and the newly proposed staging system. The adjusted hazard ratios for the new IB, IIA, IIB, and III, with stage IA as reference, are 4.311 (95% CI: 3.217-5.778), 8.993 (95% CI: 6.637-12.186), 13.179 (95% CI: 9.435-18.407), and 20.693 (95% CI: 13.655-31.356), respectively (all p-values < 0.001). In addition, the adjusted variables are shown in **Supplementary Table 3**. The cancer-specific mortality rates per 1000-person-year for the new stage IA, IB, IIA, IIB and III were 0.812 (95% CI: 0.674-0.978), 6.612 (95% CI: 5.936-7.364), 22.228 (95% CI: 20.128-24.547), 50.863 (95% CI: 47.472-54.496) and 120.318 (95% CI: 112.596-128.570), respectively (**Table 3**). ROC curve was shown in **Figure 6** with area under the curve of 0.908 and P-value < 0.001. Calibration curve were shown in **Figure 7**, which nearly overlapped with the calibration, with results of Hosmer and Lemeshow Test showing in **Supplementary Table 4**. Furthermore, comparison of 1000-person-year between the 8th edition and the new proposed TNM staging system, and the results of the COX analyses and 1000-person-yearof adjusted distribution are shown in **Table 4** and **Supplementary Table 5**.

**Table 3:** Comparison of the differences of the adjusted* Cox analysis of cancer specific mortality in patients with cutaneous melanoma between the 8^th^ edition and new proposed TNM staging system.

Stage	8^th^ edition		New proposed	
	HRs (95% CI)	P-value	HRs (95% CI)	P-value
IA	Ref		Ref	
IB	4.106 (3.065-5.500)	<0.001	4.311 (3.217-5.778)	<0.001
IIA	7.705 (5.658-10.492)	<0.001	8.993 (6.637-12.186)	<0.001
IIB	8.879 (6.441-12.238)	<0.001	13.179 (9.435-18.407)	<0.001
IIC	10.067 (7.019-14.440)	<0.001	–	–
III	20.013 (13.460-29.754)	<0.001	20.693 (13.655-31.356)	<0.001

adjusted for age at diagnosis, race, sex, histology subtype, thickness, ulceration, selected lymph node dissection, mitotic count rate, radiation, chemotherapy method, and surgery method; HR, hazard ratio; CI, confidence interval.

**Table 4:** Comparison of mortality (per 1000-person-years) between the 8^th^ edition and the new proposed TNM staging system based on cancer-specific survival.

Stage	8^th^ edition		New proposed	
	Fail	Rate (95% CI)	Fail	Rate (95% CI)
IA	111	0.812 (0.674-0.978)	111	0.812 (0.674-0.978)
IB	331	6.612 (5.936-7.364)	331	6.612 (5.936-7.364)
IIA	273	19.417 (17.237-21.872)	392	22.228 (20.128-24.547)
IIB	332	36.839 (33.082-41.023)	810	50.863 (47.472-54.496)
IIC	317	83.210 (74.511-92.926)	–	–
III	1156	82.803 (78.157-87.725)	876	120.318 (112.596-128.570)

CI, confidence interval.

## Discussion

Our findings demonstrated that the new classification system provides a more practical, accurate and prognosis-relevant staging system for patients with cutaneous melanoma, especially those patients who classified as stage I-III. Reliable assessment of prognosis and rational treatment planning is associated with accurate staging of the melanoma. The AJCC staging system, which codes the extent of the primary tumor (T), regional lymph nodes (N), and distant metastases (M) provides a “staging grouping” based on T, N, and M, which is the most widely used among clinicians (11). Thus, the TNM staging system is significant in patients with cutaneous melanoma, as it guides their diagnosis, treatment plan, and prognosis. We found that this clinical staging system was out dated; although, some specific provisions and pathological staging had been changed (12).

In this study, we proposed a new staging system based on the 8^th^ edition of TNM staging and guidelines of treatment, which considered prognoses, MSS, and clinical practicality. This new staging system was in accordance with scientific and universal statistical methods and was based on previous clinical experience: stage IA, T1aN0M0; stage IB, T1b/T2aN0M0; stage IIA, T3-4aN0M0 and T2bN0M0; stage IIB, T1-4aN1-2M0 and T3-4bN0M0; and stage III, T1-3aN3M0 and T1-4bN1-3M0. For stage IV, there are no distinguishing features with the 8^th^ edition AJCC staging manual, which was found to have distant metastasis and worse disease process (10).

In previous studies, it was pointed out that ulceration, the presence of which indicates an unfavorable prognosis was an independent predictor of the outcome in patients with clinically localized primary cutaneous melanoma (13, 14). Furthermore, a study reported that the extent of ulceration, measured by diameter or percentage of tumor width, had potential implications regarding the prognosis, staging, and management of patients with cutaneous melanoma (15). According to this new proposed staging system, we found that patients with ulceration tended to have an unfavorable prognosis, which classified them into a high stage despite low T-, or N- stages.

Based on the analysis of MSS, patients with no ulceration and distant metastasis were downstaged in this new staging system, which would influence the selection of treatment and improve the patients’ quality of life. The newly released guidelines of treatment advocate for adjuvant treatment in patients classified into higher stages, for instance, stage III or IV (16). However, the serious adverse effects of adjuvant treatment are unavoidable. The most common adverse effects were cutaneous toxicities, such as: rash, pruritus, and vitiligo, and other less common but potentially life-threatening high-grade immune-related toxicities were nephritis, pneumonitis, and myocarditis (17, 18). Thus, compared with adjuvant treatment, surgical treatment is likely to decrease these adverse outcomes in patients.

Though MSS is considered as the main reference to classify patients in this staging system, we also considered clinical factors. Furthermore, although the survival rate of patients with stage T1-2aN1M0 was close to that of IIA, we classified T1-2aN1M0 into IIB to conform with IIA which only included the N0-stage. Furthermore, patients with stage IIB disease were supposed to receive surgical adjuvant treatments. The results of previous studies have revealed no clear survival benefit in patients who underwent lymph node dissection compared with those who did not; however, the treatment of patients with lymph node metastasis differs from that of those without lymph node metastasis with regard to the provision of lymph node dissections (19, 20). Thus, owing to the necessity of differential treatment, we divided stage I into IA and IB stage and treated T1-2aN1M0 as stage IIB, which needed adjuvant treatment and potential surgical treatment.

There are limitations to this new proposed staging system. The 8^th^ edition of the AJCC staging manual removed the prognosis factor of mitosis, although it was an independent predictor of melanoma (21). We did not consider this element. Treatments and diagnosis of sentinel lymph node still in controversies. In some medical center, select lymph node detect was probably not performed in patients who were supposed to receive such practice in theory. Thus, there may be errors in data collection, which leads to inaccuracy experiment results. However, we have included enough cases, about 68, 681 patients, to reduce the risk of such error in this study. Furthermore, territories, genetic, and biological factors also play a role in the development of cutaneous melanoma. However, the influence of these factors is still controversial. Thus, we followed the mainstream guideline to develop this new staging system, which suits most individuals. Improving this new staging system by including components related to the depth of the melanoma to provide a suitable staging model for clinical use are required.

In conclusion, this newly proposed staging system, which classified patients based on CSS and adjusted with previous clinical treatment experience, aimed to provide a staging model for patients with cutaneous melanoma. This model might be better for clinical practice and prognostic prediction.

## Data Availability Statement

The datasets presented in this study can be found in online repositories. The names of the repository/repositories and accession number(s) can be found below: SEER Database.

## Author Contributions

All authors contributed to the design of the study and writing of the manuscript. WW, CZ, LZ, and DC undertook the research. YH, SC, and WZ performed the analyses and interpretation of data. DH and ZL wrote the main manuscript text and prepared the figures. LG and YPW revised the article critically for important intellectual content and final approval of the version to be submitted. All authors reviewed the manuscript.

## Conflict of Interest

The authors declare that the research was conducted in the absence of any commercial or financial relationships that could be construed as a potential conflict of interest.

## Publisher’s Note

All claims expressed in this article are solely those of the authors and do not necessarily represent those of their affiliated organizations, or those of the publisher, the editors and the reviewers. Any product that may be evaluated in this article, or claim that may be made by its manufacturer, is not guaranteed or endorsed by the publisher.
