# Supplementary material for: Assessment of the Novel, Practical, and Prognosis-Relevant TNM Staging System for Stage I-III Cutaneous Melanoma
Source: Front Oncol. 2022 Apr 29;12:738298. doi: 10.3389/fonc.2022.738298 (PMC9104117; doi:10.3389/fonc.2022.738298)
Supplement: Supplementary file 1 [file Table_1.docx]

Supplement table 1: Demographics and clinical characteristics of 68861 patients with cutaneous melanoma.

| Variables | No. (%) |
| --- | --- |
| Age at diagnosis, mean (SD) | 58.48(16.20) |
| Sex |  |
| Female | 31321(45.48) |
| Male | 37540(54.52) |
| Race |  |
| White | 63788(98.64) |
| Black | 283(0.44) |
| Other | 599(0.92) |
| Year of diagnosis |  |
| 2010-2012 | 31543(45.81) |
| 2013-2015 | 37318(54.19) |
| T categories |  |
| T1a | 41142(59.75) |
| T1b | 7363(10.69) |
| T2a | 8330(12.10) |
| T2b | 1965(2.85) |
| T3a | 3354(4.87) |
| T3b | 2510(3.65) |
| T4a | 1582(2.30) |
| T4b | 2615(3.80) |
| N categories |  |
| N0 | 64059(93.00) |
| N1 | 2732(4.00) |
| N2 | 1399(2.00) |
| N3 | 671(1.00) |
| Histology |  |
| Unclassified | 38647(56.12) |
| Nodular melanoma | 5015(7.28) |
| Superficial spreading melanoma | 24457(35.52) |
| Acral lentiginous melanoma | 742(1.08) |
| Thickness, mean (SD), mm | 1.09 (1.40) |
| Ulceration |  |
| Yes | 8755(12.76) |
| None | 59869(87.24) |
| Mitotic index |  |
| 0 | 24089(48.30) |
| >=1 | 25783(51.70) |
| Selected lymph node meets |  |
| Clinical (-) and pathologic (-) | 58416(93.48) |
| Clinical (-) and pathologic (+) | 2841(4.55) |
| Clinical (+) and pathologic (+) | 1236(1.97) |
| Extension |  |
| Yes | 60137(88.46) |
| None | 7842(11.54) |
| Radiation |  |
| Yes | 624(0.91) |
| None | 68204(99.09) |
| Chemotherapy |  |
| Yes | 539(0.78) |
| None | 68322(99.22) |
| Surgery method |  |
| None | 2620(3.81) |
| Destruction | 7574(11.02) |
| Excision | 58508(85.16) |

Abbreviations: SD, standard deviation; a Standard deviation

Supplement table 2: Patient distributions and per 1000-person-year based on cancer-specific mortality of 32 groups

| Code | Groups | CSS (fail) | OS (fail) | 1000-person-year (95% CI) |
| --- | --- | --- | --- | --- |
| 1 | T1aN0M0 | 40863(111) | 40863(1693) | 0.812(0.674-0.978) |
| 2 | T1bN0M0 | 6983(147) | 6983(535) | 6.007(5.111-7.061) |
| 3 | T2aN0M0 | 7407(184) | 7407(589) | 7.189(6.222-8.307) |
| 4 | T2bN0M0 | 1627(107) | 1627(266) | 20.223(16.703-24.286) |
| 5 | T3aN0M0 | 2659(166) | 2659(419) | 18.939(16.266-22.050) |
| 6 | T3bN0M0 | 1815(213) | 1815(490) | 39.272(34.337-44.917) |
| 7 | T4aN0M0 | 1167(119) | 1167(273) | 33.161(27.708-39.688) |
| 8 | T4bN0M0 | 1538(317) | 1538(655) | 83.210(74.511-92.926) |
| 9 | T1aN1M0 | 171(22) | 171(28) | 38.450(25.318-58.395) |
| 10 | T1bN1M0 | 255(41) | 255(57) | 50.713(37.199-69.137) |
| 11 | T2aN1M0 | 659(56) | 659(76) | 25.005(19.243-32.491) |
| 12 | T2bN1M0 | 196(39) | 196(53) | 66.327(48.460-90.780) |
| 13 | T3aN1M0 | 442(59) | 442(78) | 41.051(31.806-52.983) |
| 14 | T3bN1M0 | 383(80) | 383(110) | 65.749(52.811-81.857) |
| 15 | T4aN1M0 | 170(38) | 170(51) | 75.223(54.735-103.379) |
| 16 | T4bN1M0 | 456(146) | 456(207) | 127.725(108.600-150.218) |
| 17 | T1aN2M0 | 80(14) | 80(22) | 52.484(31.084-88.617) |
| 18 | T1bN2M0 | 92(20) | 92(28) | 68.985(44.506-106.928) |
| 19 | T2aN2M0 | 216(24) | 216(33) | 32.524(21.800-48.524) |
| 20 | T2bN2M0 | 105(32) | 105(40) | 111.143(78.598-157.165) |
| 21 | T3aN2M0 | 174(28) | 174(39) | 54.422(37.576-78.820) |
| 22 | T3bN2M0 | 218(76) | 218(94) | 128.541(102.660-160.947) |
| 23 | T4aN2M0 | 151(39) | 151(57) | 99.087(72.100-136.176) |
| 24 | T4bN2M0 | 363(157) | 363(187) | 187.575(160.414-219.335) |
| 25 | T1aN3M0 | 28(11) | 28(14) | 140.127(77.603-253.029) |
| 26 | T1bN3M0 | 33(17) | 33(20) | 195.402(121.474-314.323) |
| 27 | T2aN3M0 | 48(13) | 48(18) | 94.891(55.099-13.419) |
| 28 | T2bN3M0 | 37(19) | 37(21) | 222.006(141.607-348.051) |
| 29 | T3aN3M0 | 79(23) | 79(26) | 117.848(78.313-177.341) |
| 30 | T3bN3M0 | 94(34) | 94(46) | 138.917(99.260-194.418) |
| 31 | T4aN3M0 | 94(49) | 94(55) | 228.803(171.910-304.525) |
| 32 | T4bN3M0 | 258(119) | 258(149) | 248.089(207.299-296.918) |

Abbreviation: CI, confidence interval

Supplement table 3: Adjusted Cox analysis of cancer specific mortality of patients with cutaneous melanoma

| Variables |  | HRs (95%CI) | P-value |
| --- | --- | --- | --- |
| Age at diagnosis | | 1.025(1.021-1.028) | <0.001 |
| Sex | Female | Ref |  |
|  | Male | 1.314(1.182-1.461) | <0.001 |
| Race | White | Ref |  |
|  | Black | 1.576(1.086-2.288) | 0.017 |
|  | Other | 1.066(0.709-1.602) | 0.759 |
| Year of diagnosis | 2010-2012 | Ref |  |
|  | 2013-2015 | 0.777(0.695-0.868) | <0.001 |
| Histology | Unclassified | Ref |  |
|  | Nodular melanoma | 1.195(1.063-1.350) | 0.003 |
|  | Superficial spreading melanoma | 0.936(0.818-1.070) | 0.333 |
|  | Acral lentiginous melanoma | 1.330(1.035-1.710) | 0.026 |
| Thickness |  | 1.001(1.001-1.001) | <0.001 |
| Ulceration | Yes | Ref |  |
|  | None | 1.334(1.144-1.556) | <0.001 |
| Mitotic index | 0 | Ref |  |
|  | >=1 | 2.111(1.671-2.666) | <0.001 |
| Select lymph node dissection | Clinical (-) and pathologic (-) | Ref |  |
|  | Clinical (-) and pathologic (+) | 1.444(1.167-1.787) | 0.001 |
|  | Clinical (+) and pathologic (+) | 1.960(1.559-2.465) | <0.001 |
| Extension | Yes | Ref |  |
|  | None | 1.206(1.065-1.366) | 0.003 |
| Stage | IA | Ref |  |
|  | IB | 4.303(3.211-5.766) | <0.001 |
|  | IIA | 8.993 (6.637-12.186) | <0.001 |
|  | IIB | 13.179(9.435-18.407) | <0.001 |
|  | III | 22.713(14.835-34.775) | <0.001 |
| Radiation | Yes | Ref |  |
|  | None | 1.728(1.432-2.086) | <0.001 |
| Chemotherapy | Yes | Ref |  |
|  | None | 1.522(1.228-1.887) | <0.001 |
| Surgery method | None | Ref |  |
|  | Destruction | 0.753(0.491-1.154) | 0.193 |
|  | Excision | 0.463(0.311-0.689) | <0.001 |

Abbreviation: HR, hazard ratio; CI, confidence interval

Supplement table 4: Contingency table of Hosmer and Lemeshow Test for the new proposed staging model

|  | MSS = 0 | | MSS = 1 | |  |
| --- | --- | --- | --- | --- | --- |
| Group | Observed | Expected | Observed | Expected | total |
| 1 | 4148 | 4146.691 | 2 | 3.309 | 4150 |
| 2 | 4149 | 4144.477 | 1 | 5.523 | 4150 |
| 3 | 4145 | 4142.734 | 6 | 8.266 | 4151 |
| 4 | 4141 | 4137.196 | 9 | 12.804 | 4150 |
| 5 | 4134 | 4130.272 | 16 | 19.728 | 4150 |
| 6 | 4119 | 4116.364 | 31 | 33.636 | 4150 |
| 7 | 4081 | 4085.846 | 69 | 64.154 | 4150 |
| 8 | 4025 | 4015.283 | 125 | 134.717 | 4150 |
| 9 | 3835 | 3854.377 | 315 | 295.623 | 4150 |
| 10 | 3115 | 3118.760 | 1029 | 1025.240 | 4144 |

Supplement table 5: Adjusted Cox analysis and per 1000-person-year of cancer-specific mortality based on the categories of adjusted distribution

| Stage | | Cox analysis | | 1000-person-year | |
| --- | --- | --- | --- | --- | --- |
|  | HRs (95% CI) | | P-value | Fail | Rate (95% CI) |
| I | Ref | |  | 442 | 2.366(2.156-2.598) |
| IIA | 3.252(2.698-3.920) | | <0.001 | 470 | 22.989(20.998-25.169) |
| IIB | 4.295(3.393-5.436) | | <0.001 | 732 | 55.844(51.933-60.048) |
| IIIA | 6.419(4.569-9.017) | | <0.001 | 687 | 107.568(99.802-115.939) |
| IIIB | 7.400(4.936-11.095) | | <0.001 | 189 | 210.702(182.706-242.988) |

Abbreviation: adjusted for age at diagnosis, race, sex, histology subtype, thickness, ulceration, selected lymph node dissection, mitotic index, radiation, chemotherapy method, and surgery method; HR, hazard ratio; CI, confidence interval
